# Perioperative Multidisciplinary Intervention Led to Complete Minimally Invasive Transthoracic Esophagectomy for a Patient With Severe Lung Dysfunction: A Case Report

**DOI:** 10.7759/cureus.97931

**Published:** 2025-11-27

**Authors:** Makoto Matsumoto, Masashi Hashimoto, Kento Kawasaki, Tomoyoshi Kunitomo, Naoaki Maeda, Shunsuke Tanabe, Kazuhiro Noma, Toshiyoshi Fujiwara

**Affiliations:** 1 Department of Gastroenterological Surgery, Okayama University Graduate School of Medicine, Dentistry, and Pharmaceutical Sciences, Okayama, JPN

**Keywords:** copd, esophagectomy, perioperative multidisciplinary intervention, perioperative rehabilitation, respiratory function training and rehabilitation, sarcopenia, severe pulmonary dysfunction

## Abstract

Risk factors for postoperative pneumonia after esophagectomy include smoking, severe lung dysfunction, and sarcopenia. Heavy smokers often have chronic obstructive pulmonary disease (COPD), which is associated with poor physical activity and low muscle strength. Sarcopenia is also associated with decreased physical function and malnutrition. These factors lead to a close relationship between COPD and sarcopenia. This report describes the case of a 74-year-old man who presented with dysphagia and was diagnosed with advanced esophageal cancer with lymph node metastasis. Preoperative respiratory function testing showed a forced expiratory volume in one second (FEV1) of 0.76 L because of his past smoking and COPD. Multidisciplinary intervention was started, along with neoadjuvant chemotherapy. Preoperative management improved his physical function. Robot-assisted thoracoscopic subtotal esophagectomy with the patient in the prone position was performed with curative resection and no severe postoperative complications. The perioperative multidisciplinary intervention improved physical functions and enabled safe robot-assisted thoracoscopic esophagectomy for the patient with severe lung dysfunction in the prone position. This case highlights that not only respiratory status but also physical parameters should be taken into account when considering whether a patient can tolerate surgery safely.

## Introduction

The risk of esophageal cancer is related to various underlying conditions, and many patients who are diagnosed already have poor nutritional status or respiratory diseases. Postoperative pneumonia after esophagectomy is linked to smoking, low respiratory function, and sarcopenia, which is associated with chronic obstructive pulmonary disease (COPD), inactivity, malnutrition, and neoadjuvant chemotherapy (NAC) [1,2]. Both conditions, COPD and sarcopenia, are reversible through multidisciplinary intervention [3,4].

The Perioperative Management Center in Okayama University (PERIO) aims to efficiently provide a safe, sure, and comfortable environment perioperatively. PERIO starts interventions targeting nutrition, respiratory function, and physical function from the outpatient stage to reduce complications and improve overall survival (OS) in high-risk patients [5,6].

Here, we report a case of advanced esophageal cancer with severe pulmonary dysfunction successfully managed with multidisciplinary interventions targeting nutrition, respiratory, and physical function, enabling completion of robot-assisted thoracoscopic surgery.

## Case presentation

**Figure 1:**
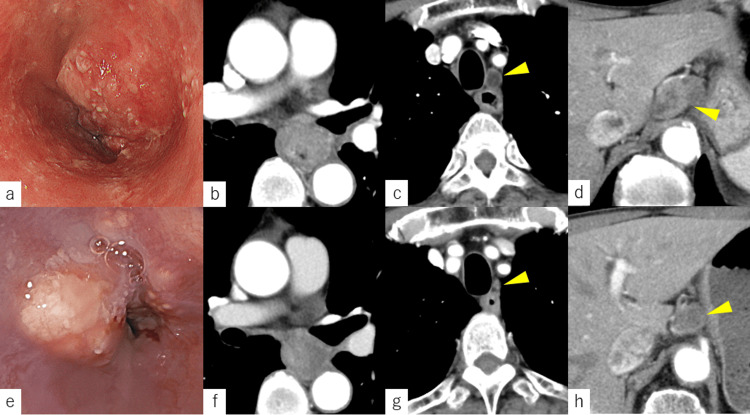
Images from upper gastrointestinal endoscopy and computed tomography (CT) Before NAC: (a) Upper gastrointestinal endoscopy shows an advanced tumor with stenosis; (b) CT shows the advanced cancer in the middle thoracic esophagus; (c) CT shows the lymph node metastasis around the left recurrent laryngeal nerve; (d) CT shows the abdominal lymph node metastasis. After NAC: (e) Upper gastrointestinal endoscopy shows that the stenosis remains; (f) CT shows that the tumor appears decreased; (g) CT shows that the lymph node metastasis around the left recurrent laryngeal nerve is significantly smaller; (h) CT shows that the abdominal lymph node metastasis has shrunk slightly.

A 74-year-old man complaining of dysphagia was referred to our hospital. He was a past heavy smoker (40 cigarettes/day for 40 years). Upper gastrointestinal endoscopy showed an ulcerated tumor with stenosis (Figure 1a). The pathological examination revealed squamous cell carcinoma. Computed tomography (CT) and 8F-fluorodeoxyglucose (FDG) positron emission tomography (PET)/CT suggested two regional lymph node metastases in the upper mediastinum and abdomen, but no distant metastasis (Figures 1b-1d). Therefore, the patient was diagnosed with esophageal cancer (cT3N1M0, cStage IIIA, Union for International Cancer Control (UICC) staging manual, 8th edition [7]).

Respiratory function testing at admission showed a forced expiratory volume in one second (FEV1) of 0.76 L, vital capacity (VC) of 2.48 L, and FEV1 as a percentage of the predicted value (FEV1%) of 35.2%. Furthermore, grip strength was 19.6 kg, skeletal muscle index (SMI) was 5.2 kg/m^2^, 6-m walk speed was 1.15 m/s, and he was diagnosed with sarcopenia [8].

Definitive chemoradiotherapy (CRT) or surgery following NAC was considered. Given the tumor size, location, and the presence of metastases, in the upper mediastinum and abdomen, it was believed that CRT alone might not achieve sufficient therapeutic effect.

**Figure 2:**
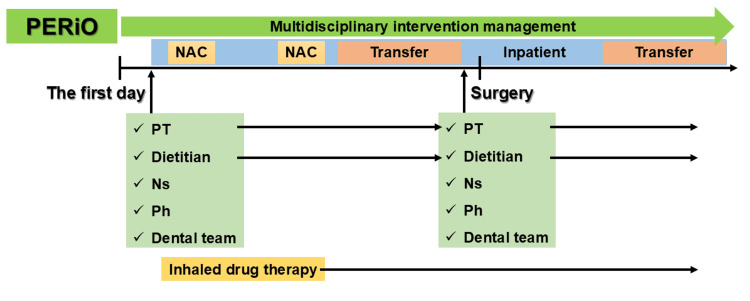
Multidisciplinary intervention schedule PERiO: Perioperative Management Center in Okayama University; NAC: neoadjuvant chemotherapy; PT: physical therapist; Ns: nurse; Ph: pharmacist

Multidisciplinary intervention through PERiO, consisting of anesthesiologists, surgeons, dedicated nurses, physical therapists, pharmacists, nutritionists, and dentists, was initiated prior to NAC (Figure 2). Physical rehabilitation, respiratory rehabilitation using volume-oriented incentive spirometry (VIS) (Coach 2® incentive Spirometer; ICU Medical (formerly Smiths Medical), San Clemente, California, United States). The patient was started on inhaled drug therapy with tiotropium bromide hydrate, a long-acting muscarinic antagonist (LAMA), and a long-acting beta agonist (LABA). He had dysphagia due to the advanced esophageal cancer, and he could not eat enough. The dietitians adjusted the form and amount of the diet and added oral nutritional supplements to meet the patient’s energy needs, which were targeted at 1,400 kcal and 55 g protein.

Fortunately, the performance status (PS) was good, and physical function was preserved, so NAC was introduced. The patient underwent two cycles of NAC with docetaxel (70 mg/m2), cisplatin (70 mg/m2), and 5-fluorouracil (700 mg/m2) (DCF) every four weeks.

After NAC, the primary tumor and the lymph node metastases showed shrinkage (Figures 1e-1h). The patient was transferred to another hospital and continued rehabilitation and inhaler therapy. Preoperatively, FEV1 was 0.84 L, grip strength was 22.3 kg, SMI was 5.7 kg/m^2^, and 6-m walk speed was 1.43 m/s (Table 1). Although he still had low lung function, his physical function had improved.

**Table 1:** General condition before and after multidisciplinary intervention in PERIO *Reference values calculated based on the patient's age, sex, nationality, and height; ** Reference values from Asian Working Group for Sarcopenia: 2019 consensus update on sarcopenia diagnosis and treatment [8]; ^†^ Reference values based on literature predicting prognosis in esophageal cancer [9] FEV1: Forced expiratory volume in one second; FEV1%: FEV1 as a percentage of the predicted value; VC: vital capacity; SMI: Skeletal muscle index; PNI: prognostic nutritional index; PERIO: Perioperative Management Center in Okayama University

Parameters	Reference data	Patient Results Before Intervention	Patient Results After Intervention
Respiratory function			
FEV1 (L)	>2.65*	0.76	0.84
FEV1% (%)	>80*	35.2	28.9
VC (L)	>3.4*	2.48	2.74
Physical function			
Grip strength (kg)	>28**	19.6	22.3
6-m walk speed (m/s)	>0.8**	1.15	1.43
SMI (kg/m^2^)	>7.0**	5.2	5.7
6-min walk distance (m)	600^†^	360	510
Body mass (kg)	61.5*	39.3	41.1
Body mass index (kg/m^2^)	21.5~24.9*	15.2	15.9
Nutritional status			
PNI	>40^†^	51.9	42.4

**Figure 3:**
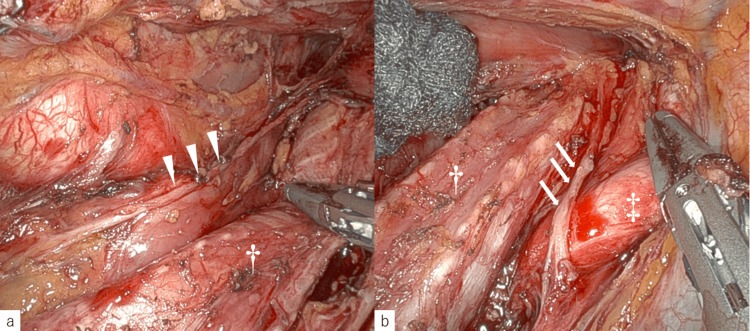
Surgical findings of esophagectomy †: bronchus, ‡: right subclavian artery (a) The lymph node metastasis around the left recurrent laryngeal nerve (arrowheads) is sufficiently dissected; (b) Right upper mediastinal dissection around the right recurrent laryngeal nerve (arrows) is performed the same as on the left.

Both the mediastinoscopic approach and the robot-assisted thoracoscopic approach with the patient in the prone position were considered. However, robot-assisted thoracoscopic surgery with continuous neuro integrity monitoring (NIM) was selected because sufficient lymph node dissection was necessary for curative resection without recurrent laryngeal nerve paralysis. If severe acidosis associated with uncontrollable hypercapnia occurred before dissecting the mediastinal pleura, switching to mediastinoscopy was planned. The artificial pneumothorax pressure was adjusted to 8-10 mmHg using carbon dioxide (CO_2_). The maximum end-tidal CO_2_ was 44 mmHg, and the maximum arterial CO_2_ was 64.9 mmHg. Therefore, the thoracoscopic surgery in the prone position was succussed with no complication (total operation time: 533 minutes, thoracic part: 165 minutes, blood loss:30 ml) (Figure 3).

All multidisciplinary interventions resumed the day after surgery. No severe complications, including respiratory complications, were observed postoperatively, and the patient was transferred to another hospital on postoperative day 27. He continued rehabilitation and was discharged home 85 days after the surgery.

The pathological diagnosis was well-differentiated squamous cell carcinoma pT3N1M0, pStage III, UICC 8th edition [7]. There was one metastasis in the left recurrent nerve lymph nodes. Four months postoperatively, local recurrence and multiple liver, lung, and bone metastases were observed immediately after discharge. The patient has been receiving chemotherapy in the outpatient setting for 11 months after the surgery so far.

## Discussion

This report presented a case of advanced esophageal cancer with decreased lung function and risks of postoperative complications. Multidisciplinary intervention was performed before NAC to prevent complications and perform curative surgery. Active rehabilitation interventions, along with accurate evaluations of nutrition, respiratory function, and physical function, contributed to a safe robot-assisted esophagectomy without complications.

COPD is associated with multiple systemic conditions, such as airway obstruction, malnutrition, and low muscle mass, and COPD and sarcopenia affect each other [10]. Sarcopenia causes poor outcomes, with gait speed being an important factor [11,12]. Improvement of sarcopenia requires a multifaceted approach [3]. On the physical rehabilitation, Ikeda et al. describe how rehabilitation during NAC can prevent loss of grip strength and reduce postoperative complications [13]. Of note, rehabilitation may allow for adequate completion of NAC. Furthermore, the other study reported that intervening before NAC may prevent adverse events and provide safer perioperative management [14]. Although patients with severe lung dysfunction and preoperative sarcopenia are at high risk for discontinuation of chemotherapy and dose reduction, these supports achieved complete NAC-DCF without serious adverse events in this case.

Some reports indicate that synergistic effects of multidisciplinary interventions, including nutritional management and physical rehabilitation, as well as inhaled medication, lead to a reduction in postoperative complications [15-17]. Both nutritional and rehabilitation interventions are necessary to improve physical function and muscle strength [15,16]. LAMA and LABA combination therapy is effective at improving physical function in COPD patients [17]. COPD patients who received rehabilitation combined with LAMA and LABA showed a reduction in postoperative complications and improved long-term prognosis compared to those who received rehabilitation alone [18]. Although some parameters, such as the prognostic nutritional index (PNI) and FEV1, did not improve numerically in this case, the concurrent implementation of multidisciplinary interventions may have produced a synergistic effect, improving physical function and facilitating a safe surgical procedure.

In the present case, physical and respiratory rehabilitation using VIS was started. Incentive spirometry encourages patients to take deep breaths and provides visual feedback. It is used to improve pulmonary function and prevent pulmonary complications. Incentive spirometry includes flow-oriented incentive spirometry (FIS), targeting inspiratory flow, and VIS, targeting inspiratory volume. In a randomized controlled trial (RCT), VIS improved respiratory function more than FIS [19]. It has been reported that postoperative pulmonary complications were prevented in esophagectomy patients who received respiratory rehabilitation using VIS; the report suggests that improving overall muscle strength can also enhance respiratory muscles [20].

Regarding surgical procedures, thoracoscopic surgery and mediastinoscopic surgery were considered. Thoracoscopic esophagectomy is approached from the right thoracic cavity, which allows dissection of large or advanced cancers from outside of the pleura. In contrast, mediastinoscopy is a safe procedure even for patients with low lung function because of two-lung ventilation. Considering the approach from the narrow intramediastinal space, there are some difficulties, the risk of recurrent laryngeal nerve palsy or radial margin-positives, and the number of dissected lymph nodes [21]. In patients with low lung function, aspiration due to recurrent laryngeal nerve paralysis can be fatal, and adequate lymph node dissection is necessary for advanced esophageal cancers. Therefore, we planned robot-assisted surgery using NIM.

Multidisciplinary management was performed to focus on three points. First, physical and respiratory rehabilitation improved the patient’s muscle strength, including the respiratory muscles. Second, dietitians supported the nutritional management of the patient. Finally, inhalers for severe COPD increased his lung function. Although respiratory function and nutritional scores did not improve on examination, physical function significantly improved. While all interventions had a synergistic effect, in this case, the improvement in physical function contributed most significantly to achieving the safest surgical outcome. As limitations of this report, the criteria for safe thoracoscopic esophagectomy, such as respiratory function and sarcopenia, are still unclear. Such cases should be performed at facilities with extensive experience in esophageal cancer surgery with perioperative management teams.

## Conclusions

Although multidisciplinary intervention during neoadjuvant chemotherapy did not improve respiratory function or nutritional parameters, the patient’s physical function showed marked improvement. Such case reports are rare, and it is difficult to establish absolute reference values. This case highlights the importance of comprehensive patient assessment that includes not only pulmonary function tests but also physical performance and nutritional indicators.
